# Autogenous Tooth Bone Grafts for Repair and Regeneration of Maxillofacial Defects: A Narrative Review

**DOI:** 10.3390/ijerph19063690

**Published:** 2022-03-20

**Authors:** Omer Sefvan Janjua, Sana Mehmood Qureshi, Muhammad Saad Shaikh, Ahmad Alnazzawi, Francisco J. Rodriguez-Lozano, Maria Pilar Pecci-Lloret, Muhammad Sohail Zafar

**Affiliations:** 1 Department of Maxillofacial Surgery, PMC Dental Institute, Faisalabad Medical University, Faisalabad 38000, Pakistan; osj1982@hotmail.com; 2 Department of Oral Pathology, PMC Dental Institute, Faisalabad Medical University, Faisalabad 38000, Pakistan; sana.mehmood@outlook.com; 3 Department of Oral Biology, Sindh Institute of Oral Health Sciences, Jinnah Sindh Medical University, Karachi 75510, Pakistan; drsaadtanvir@gmail.com; 4 Department of Substitutive Dental Sciences, College of Dentistry, Taibah University, Al Madinah al Munawwarah 41311, Saudi Arabia; alnazzawi@gmail.com; 5 Gerodontology and Special Care Dentistry Unit, Hospital Morales Meseguer, Medicine School, University of Murcia, 30100 Murcia, Spain; mariapilar.pecci@um.es; 6 Department of Restorative Dentistry, College of Dentistry, Taibah University, Al Madinah al Munawwarah 41311, Saudi Arabia; mzafar@taibahu.edu.sa; 7 Department of Dental Materials, Islamic International Dental College, Riphah International University, Islamabad 44000, Pakistan

**Keywords:** bone grafting, dental implants, regeneration, alveolar bone loss

## Abstract

Autogenous tooth graft is an innovative and ingenious technique that employs a stepwise approach and utilizes human teeth as an autogenous source of bone graft. The structure of teeth closely resembles bone, both physically and biochemically, and can be efficiently used for the process as it depicts properties of osteoinduction and osteoconduction. Autogenous tooth bone has characteristics similar to bone grafts in terms of healing potential, physical properties, and clinical outcome. Autogenous tooth graft has shown reasonable promise as a graft material for the regeneration of maxillary and mandibular defects. Autogenous tooth bone graft finds its principal application in sinus and ridge augmentations and for socket preservation before implant placement. Additionally, it can be used successfully for alveolar cleft patients and patients with limited periodontal defects. The overall complication rates reported for autogenous tooth grafts are comparable to other graft sources. However, although long-term results are still underway, it is still recommended as a grafting option for limited defects in the cranio-facial region.

## 1. Introduction

Bone loss in the craniofacial region occurs following traumatic tooth extraction, trauma, pathologies, or due to periodontal diseases [1,2]. Replacement and regeneration of the lost bone require grafting. For maxillofacial applications, there is a wide range of sources and bone grafting materials (Table 1) categorized as autogenous, allogenic, xenogenic, alloplastic, and engineered personalized grafts [3,4]. Autogenous bone is derived from some other donor site of the same individual and renders the properties of osteogenesis (the formation of new bone by the graft cells), osteoinduction (the recruitment and stimulation of undifferentiated pluripotent cells into bone-forming cells), and osteoconduction (the provision of a scaffold which allows new bone formation over it) [5,6,7]. However, these beneficial properties are not without demerits, the biggest being the need for a donor site morbidity [8,9]. Allogenic bone is acquired from another individual of the same species and includes freeze-dried bone allograft (FDBA), demineralized freeze-dried bone allograft (DFDBA), lyophilized bone, etc. The advantage is that there is no need for donor site surgery; however, the property of osteogenesis is lost when the bone is prepared for clinical use. The property of osteoinduction is retained to some extent but requires a carrier to meet the clinical requirements [10,11]. Xenogenic bone is acquired from other species, the most common ones being bovine or porcine sources. It also shares the same advantages and disadvantages as mentioned for the allogenic bone [10,12]. Lastly, the alloplastic bone refers to commercially prepared bone (usually in the form of calcium phosphate/tri-calcium phosphate). Again, alloplastic bone, like allogenic and xenogenic bone, allows only osteoconduction to occur at the grafting site [13,14]. Engineered bone grafts that utilize mesenchymal stem cells, scaffolds, and biologically active factors or molecules to regenerate bone, can overcome these problems in the future; however, the technique is still in infancy and their chairside/commercial preparation is still not widespread [15,16].

All the literature and evidence so far suggest that the autogenous bone source is the best; hence, the quest continues for the acquisition of autogenous bone with minimum donor site morbidity [4,17]. Autogenous tooth bone graft is a type of autogenous grafting technique that utilizes the extracted tooth/teeth of the same individual for the preparation of the graft material. Extracted teeth of the same individual provide such a valuable source without causing any harm since teeth have already been extracted, hence the need for a donor site surgery is eliminated [18]. Although the use of autogenous tooth bone graft (AUTO-BG) was first described in 2003, it still remains a less commonly used technique in clinical practice. Therefore, the aim of this review paper is to discuss the AUTO-BG, its method of preparation, utilities, and applications in the jaw region and associated limitations.

**Table 1:** Different sources of graft with their individual properties.

Type	Available Sources	Advantages	Disadvantages
Autogenous [19,20,21]	Extra-oral sites: Crest of the iliac bone, tibia, parietal bone, ribs, sternum. Intra-oral sites: Mandibular symphysis, ramus, maxillary tuberosity, zygomatic buttress, extraction socket, coronoid process, autogenous tooth.	Potential for osteogenesis, osteoinduction, osteoconduction, and osteopromotion. No allergenic and immune-mediated reaction and no possibility of graft rejection. Low cost.	Donor site surgery and morbidity. Increased surgical time which may require general anesthesia. Very large quantities cannot be harvested without significant donor site deficit.
Allogenic [21]	FDBA DFDBA DBM	Acts as a scaffold and allows osteoconduction. No donor site surgery/morbidity. Can be combined with other materials such as BMP, GFs, PRF to enhance its healing potential.	Processing required to remove allergenic component Rejection by the host is possible.
Xenogenic [19,21] (different species)	Porcine source Bovine source Corals Algae	Acts as a scaffold and allows osteoconduction. No donor site surgery. Can be combined with other materials such as BMP, GFs, PRF to enhance its healing potential. Low cost. Significant quantities can be acquired.	Processing required to remove allergenic components but still can transmit disease. Possibility of rejection.
Alloplastic [20] (synthetically produced)	TCP *β*-TCP Bioactive Glass Bio-ceramics Hydroxyapatite	Acts as a scaffold and allows osteoconduction. No donor site surgery. Can be combined with other materials such as BMP, GFs, PRF to enhance its healing potential. No allergenic potential.	Can be costly. Can act as foreign body.
Engineered personalized bone grafts [22,23,24]	Bioactive acellular scaffolds: Biodegradable synthetic materials with osteoinductive factors such as BMPs, PDGF, IGF. Cell seeded scaffolds: Autologous BMSCs in a customized scaffold mixed with PRP. Customized autologous bone grafts: Pluripotent stem cells induced to form bone.	Autologous stem cells with decreased chances of rejection. Can be molded into the desired anatomical shape using 3D modeling. Inclusion of bioactive molecules provides a better healing potential.	Still in infancy and further research needed to bring into clinical use. Requires facilities to harvest and culture stem cells. May have ethical issues.

*β*-TCP (*β*-tri-calcium phosphate); BMPs: bone morphogenetic proteins; BMSCs: bone marrow stem cells; DDM: demineralized dentine matrix; DFDBA: demineralized freeze-dried bone allograft; FDBA: freeze-dried bone allograft; GFs: growth factors; IGF: insulin growth factors; PDGF: platelet-derived growth factor; PRF: platelet-rich fibrin; PRP: platelet-rich plasma; TCP: tri-calcium phosphate.

The reason why researchers have been advocating the use of AUTO-BG is manyfold. AUTO-BG resembles physically and biochemically an autogenous bone graft, shows biocompatibility and bioactivity like that of an autogenous bone graft, and demonstrates acceptable clinical outcomes. These characteristics are reviewed in detail in the following sections. For this purpose, PubMed, MEDLINE, EMBASE, Scopus, Web of Science, and Google Scholar were extensively searched using search strategies such as ‘Autogenous tooth’, ‘Tooth graft’, ‘Tooth bone graft’, ‘bone graft’, and ‘autogenous bone graft’. Full-text articles, review articles, case series, case reports, and abstracts were selected which matched the search criteria.

## 2. Composition and Biochemical Properties of AUTO-BG

Dentin is the main component that forms the bulk component of teeth [25]. Human dentin is composed of inorganic (55%) and organic (45%) material and shares almost the same chemical and physical properties as that of bone. The main ingredients of both of these mineralized tissues include collagenous (18%), non-collagenous proteins (2%), and mineralized content in the form of various calcium phosphates (70%) in weight volume. Demineralized dentin and bone matrices primarily comprise type I collagen (95%), while the non-collagenous proteins include dentin sialophosphoprotien (DSPP), dentin matrix protein-1, bone sialoprotein, osteopontine, and osteonectin. Demineralized matrices also contain bone morphogenetic proteins (BMPs) and fibroblast growth factors [26,27].

Dentin demineralized matrix (DDM) is an acid-resistant collagen that is absorbable and contains a micro-tubular structure and possesses a blood coagulation property. The presence of collagen I and III, DSPPs, BMPs, and TGF-β imparts osteogenic and osteoinductive influence to AUTO-BG proteins [28]. The inorganic component of AUTO-BG material contains five different types of calcium phosphates along with trace elements such as zinc, chloride, and iron. The calcium phosphates include hydroxyapatite, tri-calcium phosphate, amorphous calcium phosphate, dicalcium phosphate dehydrate, and octacalcium phosphate. The presence of these calcium phosphates allows the material to act as a scaffold (osteoconductive property) [27]. The crystallinity of enamel is even better than that of dentin with an even greater apatite content giving it stability against dissolution.

## 3. General Characteristics of AUTO-BG

AUTO-BG can be prepared either as a block type or powder type. The block type is further sub-categorized as root-form or root-on types. The root-form is used for the preservation of extraction sockets because of its shape, which resembles a tooth root. The root-on type is used for ridge augmentation (horizontal or vertical) because its shape resembles a cortical block graft [27,29,30]. A study conducted by Kim et al. showed that the crown portion of AUTO-BG is composed of a higher calcium to phosphate ratio and a high-crystalline calcium phosphate mineral (which is mainly hydroxyapatite). On the other hand, the root portion contained low-crystalline calcium phosphates and a generally low calcium to phosphate ratio [31]. This difference, as mentioned above, is due to the difference in crystallinity of enamel and dentin.

Powder-type graft materials, referred to as tooth ash by Zhang et al. [28], can be either crown type (AUTO-BG enamel) or root type (AUTO-BG dentine). The crown type comprises mostly of inorganic enamel and carries the capacity for osteoconduction and can maintain bone volume after grafting. The root type principally comprises dentine and cementum and is mostly organic and hence has the potential of osteoinduction and osteoconduction and is generally used in ridge augmentations [27,32,33].

### 3.1. Biocompatibility of AUTO-BG

Both the powder-type and block-type AUTO-BG have shown excellent biocompatibility and regeneration potential. According to Kim et al., the AUTO-BG resorbs slowly and is gradually replaced by new bone [34]. A histological study by Kim et al. showed that recipient bone and AUTO-BG material form a direct union upon graft healing [35]. On clinical evaluation at the time of implant placement, Schwarz et al. showed that grafted bone shows similar bleeding characteristics as that of the normal surrounding bone [36]. The osteoinductive potential of these odontogenic graft materials has been found to be similar to Bio-Oss and it has also been reported that it can be further enhanced by simultaneous application of platelet-rich plasma and human recombinant BMPs [28].

### 3.2. Bioactivity

Research has revealed that the dentin matrix contains BMPs, osteopontine, osteonectin, osteocalcin, dentin sialoproteins, bone connexins, and alkaline phosphatase. All these proteins have a definite role in the formation of bone and promote/maintain the calcification of bone [37]. Bessho et al. extracted BMPs from the dentine-derived matrix of human teeth and this BMP was shown to depict osteoconductive and osteoinductive potential, both in humans and in xenogenic models through BMP receptors and their downstream molecules Smad 1, 5, and 8 [38]. Similarly, Wang et al. showed that human dentin contains another protein (LIM-1) which has osteogenic potential [39]. Therefore, these studies show that human DDM not only supports new bone formation but rather potentiates its formation too. Non-collagenous proteins present in the AUTO-BG have a signaling role in new bone formation and bone remodeling. DSPP potentiates crystal formation in the apatite, osteocalcin regulates bone mineralization through activating osteoblasts, bone connexin binds minerals and collagen, osteopontine remodels bone through the induction of osteoblasts and osteoclasts, and alkaline phosphatase has a developmental role in the biomineralization of teeth and bones. In addition to these specific proteins, dentin also contains growth factors such as PDGF, VEGF, IGF, EGF, and TGF-β which mediate their effects through mesenchymal stem cells induction [40,41]. It has also been reported by Zhang et al. that DDM induces bone formation in 4 weeks, whereas partial DDM which contains around 30% mineral content takes around 8–12 weeks to show bone formation. The explanation for this finding is that demineralization exposes the BMPs contained in the matrix. VEGF promotes angiogenesis, EGF stimulates prostaglandins E2 which influence bone formation, while IGF has a direct effect on collagen production by osteoblasts [28]. The presence of all these elements in the AUTO-BG is the reason for its bioactivity.

### 3.3. Physical Properties

Kim et al. compared the surface characteristics of AUTO-BG using a scanning electron microscope and found that the physical surface characteristics were quite similar to autogenous cortical bone (obtained from mandibular buccal cortical bone). Under high magnification, the root portion of the AUTO-BG showed a rough pattern while the crown portion of AUTO-BG was relatively smooth. The compactness of the cortical bone graft was wave-like due to its cortical nature while the surface of allogenic bone was fairly smooth as it contained cancellous bone. A smaller degree of compactness was demonstrated by the xenogenic graft. In an X-ray diffraction analysis (XRD), which is a method employed to study the crystalline nature of solids, AUTO-BG showed a crystalline structure similar to that of autogenous cortical bones. The calcium and phosphorus content from the Ca/P ion dissolution test was also found to be similar to that of autogenous cortical bone. This dissolution is an indicator of biodegradability which is directly related to the release of calcium and phosphorus which is required for reprecipitation of apatite on the bone surface [42].

### 3.4. Clinical Outcome

Long-term clinical studies conducted by Lee and Kim et al. revealed excellent biocompatibility of AUTO-BG [34,43]. It was successfully depicted that AUTO-BG is resistant to infection and heals satisfactorily even with mild wound dehiscence. In another study, Kim et al. demonstrated that AUTO-BG undergoes a gradual resorption process and is ultimately replaced by good-quality bone, employing both the processes of osteoinduction and osteoconduction [34]. They demonstrated through histological samples that after 4 months of grafting, the graft material directly fuses with the recipient bone and shows excellent vascularity and according to them, the graft is completely replaced by normal bone in 12–15 months. In their study, Jun et al. showed a mean bone density of 981 HU (D2 type) in healed auto-tooth bone graft versus 968 HU for Bio-Oss. Similarly, they depicted an almost 60% proportion of new bone volume to total bone volume with AUTO-BG [44]. These studies show that the results of AUTO-BG in the maxillofacial region are comparable to other bone grafting sources.

## 4. Method of Preparation of AUTO-BG

Sound teeth that require removal are extracted using a minimally harmful approach. This protects the buccal and lingual cortical plates and thus allows a better adaptation of the graft. For producing the powder-type AUTO-BG, the extracted teeth are first thoroughly cleaned and made free from debris or any attached tissue remnants. Crown portions are separated from the root. The root portion is placed in a Smart Dentine Grinder (Kometa Bio, Fort Lee, NJ, USA) and is ground for approximately 30 s in order to produce a 300–1200 micron dentine powder. This dentin powder is then placed in a dentine cleanser for about 7 to 10 min. This dentin cleanser is a solution containing high pH (very basic) sodium hydroxide in 20% ethanol and is used to cleanse the particulate in an attempt to eliminate bacteria and any remaining organic material. Once the cleansing process is complete, the excess cleanser is removed using sterile absorbent gauzes [29]. Next, a dentine wash using a Smart Dentin Grinder (Kometa Bio, Fort Lee, NJ, USA) consisting of phosphate-buffered saline, is poured onto the particulate material for 3 min. Once the soaking is complete, the excess liquid is removed by pouring out the excess, and the rest is absorbed with gauze. After this process, the AUTO-BG is now ready for use and can be easily transferred to the recipient site like any other graft material [30,34]. In order to prepare the block-type AUTO-BG, the tooth is not subject to grinding while the rest of the process is essentially the same. Small holes may be drilled in the block-type graft in order to improve the ingrowth of vasculature in the grafted material from the recipient site. After this, it can be placed in the extraction socket for socket preservation. The root-on type resembles cortical plates and is used for vertical/horizontal augmentation of the bone [33,45].

**Figure 1:**
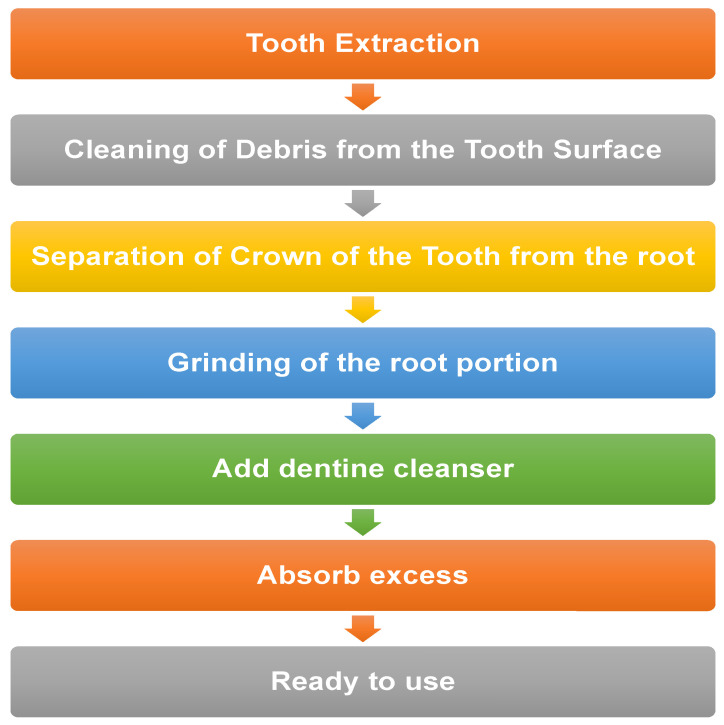
Flow chart showing method of preparation of AUTO-BG.

Powdered form or tooth ash is prepared through a high-temperature sintering process. The tooth is soaked in hydrogen peroxide to remove debris of soft tissue and then disinfected by dipping in ethanol. The tooth powder is heated for one hour at 1200 °C to remove all impurities and any remaining infected material. In order to improve its handling and placing in bone defects, it can be mixed with gypsum or platelet-rich plasma [28]. Figure 1 shows the preparation methods of AUTO-BG.

## 5. Clinical Applications of AUTO-BG

**Figure 2:**
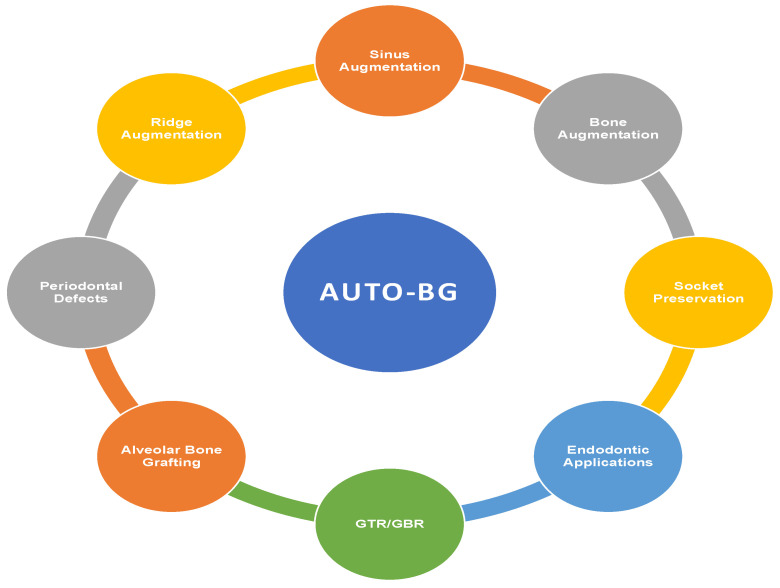
Clinical applications of AUTO-BG.

Following the development of the AUTO-BG technique and development methods, it has been explored for a range of clinical conditions in the craniofacial region (Figure 2).

### 5.1. Bone Augmentation

Dental implants are placed at an increasing number in clinical practice due to patients’ awareness and evidence supporting long-term success with almost a million implants being placed every year [46]. With this increasing number, implant surgeons are becoming increasingly confident and attempting to insert implants where the bone is inadequate in size for implants placement and in these cases, implant sites have to be grafted. Powder-type AUTO-BG is reported to be a successful bargain in such cases where osteogenic, osteoinductive, and osteoconductive properties are warranted [27,29]. Ramanauskaite et al. showed that a mean gain in alveolar ridge width was around 5 mm with an annual resorption of approximately 0.1 mm. In their study, they were able to place implants in all the grafted cases within 26 weeks of grafting with adequate primary stability [36].

In cases where the deficient bone needs to be corrected due to any reason other than implant placement, powder-type or block-type grafting, using AUTO-BG, can be employed successfully [18].

### 5.2. Sinus Augmentation

Bone resorption and sinus pneumatization in the posterior maxilla often precludes implant placement without performing sinus lift surgery [47]. Sinus lifting can be carried out either via the crestal approach or through the lateral window approach [48]. People have reported satisfactory results using various autogenous, allogenic, and/or alloplastic materials for sinus augmentation [49]. In general, any material with a slow resorption rate would work in sinus lift surgery. AUTO-BG can be regarded as a possible alternative when the autogenous bone is needed for sinus augmentation without the need for donor site morbidity. It produces a positive effect to increase the quantity and quality of bone and minimizes re-pneumatization of the sinus [45]. Kim et al. [50] showed an average increase in bone height of around 5 mm after sinus floor augmentation with AUTO-BG with an average of 0.76 mm/year bone loss after implant loading with an overall reported implant survival of around 96% in AUTO-BG sinus augmentations as reported by Shavit et al. [51].

### 5.3. Periodontal Defects

Research is being carried out in the field of bone regeneration for periodontal defects. Guided tissue regeneration (GTR) procedures [52,53,54], the use of enamel matrix derivatives (EMD) [55,56,57,58], growth factors (GF) [59,60,61], BMPs [62], platelet-rich plasma/fibrin (PRP/PRF) [63,64,65], and various bone grafting procedures [66,67,68,69] have been extensively described in the literature. Autogenous material obtained from the same individual is always considered the gold standard because of its high osteogenic, osteoinductive, and osteoconductive potential [70]. Keeping this under consideration, AUTO-BG-derived DDM and demineralized bone matrix (DBM) can provide the same beneficial effects as autogenous bone minus the need for donor site surgery and can promote bone formation in these intraosseous periodontal resorptive defects [30]. Upadhyay et al. used AUTO-BG material for the treatment of class II furcation defects and followed the cases for one year. The results of their study showed that horizontal probing depths decreased in the range of 1–2 mm and approximately 3–4 mm of bone was gained in the linear dimension [71].

A study by Indurkur et al. (2018) showed that individuals treated with AUTO-BG combined with a chorion membrane demonstrated insignificant outcomes to DFDBA with a chorion membrane in intrabony defects in all clinical outcomes, suggesting that AUTO-BG can be used as a useful alternative to DFDBA in periodontal regenerative therapy for intrabony defects. Furthermore, a case series found that AUTO-BG material with osteoinductive and osteoconductive capacities can be utilized for the treatment of intrabony defects [29].

### 5.4. Guided Bone Regeneration

Guided bone regeneration (GBR) is a process where new bone formation is guided by the use of a resorbable or non-resorbable membrane [72,73]. In most cases, GBR is performed with or prior to implant placement [73,74]. Powder-type AUTO-BG material can be placed along with an implant if the osseous defect is larger than 2 mm vertically or horizontally around the implant [45]. The use of a resorbable or non-resorbable membrane is the discretion of the surgeon. In case the operator feels that the amount of grafted material is less, it can be combined with allogenic material or PRF can be added to increase its bone-forming potential [29,33]. A study was conducted by Lee et al. where they used AUTO-BG for the purpose of GBR with and without the use of membranes. The results of their study showed that there was a net gain of bone of around 87% with no statistical difference whether the membrane was used or not [75].

### 5.5. Alveolar Bone Grafting

Alveolar bone grafting (ABG) is an important and commonly performed procedure in complete cleft lip and palate patients [76]. Various autogenous, allogenic, and alloplastic techniques for grafting have been described in the literature [30]. The use of AUTO-BG has also been reported. Authors have used both powder-type and block-type grafts in ABG. The advantage of AUTO-BG is that it can be combined with other graft materials and PRF can be placed alongside this graft in order to enhance the quality of the forming bone [77,78]. Authors have combined AUTO-BG along with distraction osteogenesis in alveolar cleft patients and have reported satisfactory results [79]. In cleft patients, the AUTO-BG can be acquired from non-functional third molars or supernumerary teeth which are quite common in cleft afflicted patients. In addition, any other tooth which must be extracted as per the orthodontic plan can also be used for this purpose [80].

In addition, pilot research intended to assess the effectiveness of an AUTO-BG in avoiding periodontal abnormalities after surgical extraction of impacted or semi-impacted lower third molars. Radiographic and periodontal evaluations of post-extractive sockets were done for this aim. The study included 10 patients, and 20 lower third molar extraction sockets were treated with a split-mouth technique. The experimental sites were filled with AUTO-BG derived from the removed lower third molars, whereas the control sites were filled with blood clot alone. Flaps were closed with the purpose of ensuring the wound’s stability. In all cases, the healing was unaffected by any problems connected with the use of the AUTO-BG. The probing pocket depth distal to the second lower molar was reduced at both surgery sites after 6 months, with a higher decrease reported at the experimental locations. Radiographic assessment also revealed that the transplanted sites had more bone gain than the control sites. The findings of this exploratory investigation show that AUTO-BG may be effective in reducing the establishment of periodontal abnormalities distal to the second lower molar after surgical extraction of the lower third molars [81].

### 5.6. Ridge Augmentation

When ridge resorption precludes the placement of the implant(s), augmentation procedures are warranted [82]. The deficiency can be in horizontal, vertical, or both dimensions [83]. In such situations, AUTO-BG can be used successfully, especially the root-on type block graft. Various clinicians have used block-type AUTO-BG material for ridge augmentations when the defects were equal to or more than 3 mm [45]. The placement of graft material in these cases helps to increase bone mass and follow-up studies have shown successful implant placement in ridges where AUTO-BG material was used [32]. Kim et al. showed through long-term follow-up studies that marginal bone resorption of around 2.5 mm occurs over a 5-year period following ridge augmentation and, in most cases, implants placed in the ridges augmented with AUTO-BG could be functionally loaded within 5–7 months [35].

### 5.7. Socket Preservation and Reconstruction

After tooth extraction, a sequence of biological cascades is set in motion which ultimately heals the extraction socket with secondary intention and in turn, leads to some degree of bone loss which is directly proportional to the trauma induced to the bone during tooth extraction [84]. Socket preservation techniques attempt to minimize this bone loss and tend to improve the quality and quantity of the bone in the healed socket. Grafting with or without a membrane has been described in the literature [85]. AUTO-BG can be used in such cases in two different ways: one being the extraction socket packed with powder-type tooth graft material and which may or may not be covered with a membrane; secondly, the root-type AUTO-BG can be used to preserve healing sockets [45]. They resemble a tooth root and can be placed in the socket and again may or may not be covered with a membrane according to the choice of the operator. Optimal healing of the sockets has been described in both conditions [30]. Radoczy-Drajko et al. demonstrated that AUTO-BG when used as a graft material for socket preservation leads to a mean bone loss of 15% in the horizontal dimension (maximum at the coronal portion and minimum at the apical) with negligible loss in the vertical dimension. These findings show that successful results can be obtained with AUTO-BG with functional restoration using implants later on [86].

### 5.8. Restorative and Miscellaneous Applications

Autogenous DDM has been reported as a potential source for the process of apexification and some authors have used it as a permanent root canal filling material. The powder-type AUTO-BG can also be used in endodontic surgery as a bone graft material to fill bony defects created by bone resorption from periapical pathology [30].

AUTO-BG may be employed as a potential grafting material for relatively small oral pathological defects such as in cases of odontogenic cyst enucleations or traumatic injuries requiring limited bone grafts [30]. As mentioned above, it can be combined with other graft sources or distraction osteogenesis procedures and can be utilized in the treatment of resected tumors or cases of pathological fractures [30]. Kizildag et al. in an animal study showed that AUTO-BG along with PRF can be used successfully to treat calvarial bone defects [87].

## 6. Complications Associated with AUTO-BG

The following complications associated with AUTO-BG have been reported in the literature [30,36,43].

### 6.1. Wound Dehiscence

Wound dehiscence has been reported as the most common complication associated with AUTO-BT grafting. Lee et al. (2013), in their case series of AUTO-BT, reported wound dehiscence in two out of nine patients. Both patients underwent horizontal ridge augmentation in the anterior mandible [43]. Similarly, Kim et al. (2014) reported wound dehiscence in one patient out of their case series of 12 patients [34]. Both authors managed dehiscence conservatively with antiseptic dressings using chlorhexidine and obtained acceptable results in the end with no significant loss of graft material. This indicates that AUTO-BT graft is susceptible to wound dehiscence and exposure but this does not necessarily mean graft failure.

### 6.2. Infection

Jeong et al. presented a case series of 51 patients and out of these, five patients presented with an infection that was initially managed with antibiotic therapy; however, two out of these five required the removal of the graft. Therefore, in their case series, the overall incidence of post-operative infection was 9.1%. This is the highest incidence of infection reported in any case series as others have reported negligible incidence of infection [78].

### 6.3. Hematoma Formation

The overall reported incidence of hematoma related to AUTO-BT grafting is 3.64%. In a case series presented by Kim et al. [34] and Lee et al. [43], there was one case each in both series that presented with hematoma formation. Both authors were able to manage their cases with pressure dressings and conservative treatment and did not require any surgical exploration or aggressive management.

### 6.4. Exposure of Fixation Screws during the Healing Period

Schwarz, in his series of cases, reported that two of his patients presented with exposure of screw head which was used to secure the graft at the time of placement which he managed conservatively and which was not associated with any significant morbidity [88].

### 6.5. Resorption of Graft in the Form of Crestal or Marginal Bone Loss

Radoczy-Drajko et al. demonstrated a bone loss of 15% in the horizontal dimension when the auto-tooth bone was used for socket preservation [85]. Kim et al. reported a marginal bone loss of approximately 2.5 mm over 5 years with reasonable success in terms of implant placement [35]. In their review paper, Cenicante et al. depicted that when AUTO-BT grafts were evaluated radiologically over 4–6 months post grafting, they showed 0.28 ± 0.13 mm bone loss in the vertical dimension and 0.15 ± 0.08mm in the horizontal dimension. However, this bone loss can be significantly higher if there is wound dehiscence in the immediate postoperative period. However, still, in all reported literature, the overall resorption is less than that reported for xenogenic or alloplastic sources, making it a suitable option [89].

### 6.6. Inability to Achieve Primary Stability while Placing Implants

In their systematic review, Gharpure et al. presented three animal-based studies where 13.04%, 4.76%, and 15.38% cases presented with a loss of primary stability, respectively. According to this review, the overall loss of primary stability was 11.6% in these three studies; however, these findings are not reported in human-based studies [90]. Most of the authors have presented excellent ISQ values at the time of implant placement (mean ISQ at implant placement 67.3) with figures improving over time (mean ISQ at second stage 75.5).

### 6.7. Failure to Achieve Osseointegration with Implant Placement

Jeong et al. [78] reported an overall implant failure rate of 3.9%, while Kim et al. [34] and Lee et al. [43] reported a 3.4% and 4.0% implant failure rate in their case series. Together, these authors placed 165 implants in 72 patients using an AUTO-BT graft and out of these 165, around six implants failed. There are case series where a 100% success rate has been reported; therefore, the mean failure rate falls to around 2.3% which is highly acceptable [30]. Park et al. showed a failure of osseointegration in two of his cases at the time of second-stage surgery but was able to place new implants immediately after removal of the failed ones [45].

### 6.8. Fracture of the Block Graft while Drilling at the Time of Fixation

Ramanauskaite in their systematic review report that there was one patient where the block graft fractured at the time of fixation with screws and had to be discarded [36].

These are almost the same complications as associated with other grafting sources which are commonly used in clinical dentistry. The rate of complications associated with auto-tooth grafts showed no statistical difference from complications reported with other sources of grafting. This makes it an equally effective grafting modality whose use should be encouraged in clinical practice.

## 7. Conclusions and Further Recommendations

AUTO-BG material is a relatively new and innovative bone graft material with all the advantages of autogenous bone owing to its very similar components to bone and can be very useful in a multitude of clinical situations. It can also address patients’ hesitation towards allograft or xenograft and provide great biocompatibility and does not cause an immune response or foreign body reaction. Moreover, the properties of osteoinduction, osteoconduction, and creeping substitution have been successfully depicted and can be manufactured in various sizes and shapes making it a promising graft material for smaller-sized defects in the maxillofacial region. However, a rather tedious process of graft acquisition, the need for a specialized dentin grinder, a limited quantity of graft that can be acquired through this process, and the unavailability of long-term data on the success of AUTO-BG limits its routine use in clinical dentistry.

Among a variety of available bone graft materials, selecting the most suitable one is challenging. Whilst choosing a graft material must be dictated by the extent of defects and procedural reasons, AUTO-BG may be considered an option given its autogenous origin and favorable clinical as well as histological outcomes when tooth extraction is required.

## Data Availability

Not applicable.
